# Superior Vena Cava Syndrome and Colon Carcinoma: A Report of a Multifactorial Association

**DOI:** 10.1155/2015/345804

**Published:** 2015-02-25

**Authors:** Joana Espírito Santo, Inês Coutinho, Ana Pimentel, Rui Garcia, Rui Marques dos Santos

**Affiliations:** ^1^Internal Medicine Department A, University Hospital Center of Coimbra, 3000-075 Coimbra, Portugal; ^2^General Surgery Department B, University Hospital Center of Coimbra, 3000-075 Coimbra, Portugal

## Abstract

*Introduction*. Superior vena cava (SVC) syndrome results from the obstruction of blood flow through the SVC, having distinct pathophysiological underlying mechanisms. Cancer is associated with an increased risk of thromboembolism that varies according to patient-, tumor-, and treatment-related factors. An individualized clinical approach is important to pursue the accurate diagnosis of the underlying pathology causing thromboembolism in cancer patients. *Case Presentation*. The authors present a case of a 58-year-old male with an infrequent presentation of an unknown colon carcinoma, who has never had any symptom until he was hospitalized with the diagnosis of superior vena cava syndrome and pulmonary thromboembolism. The patient had an advanced disease by the time of diagnosis and molecular alterations contributing to abnormal hemostasis. He presented venous and arterial thromboembolism and developed disseminated intravascular coagulopathy after surgery, anticoagulant and transfusion therapy, dying 40 days after the hospitalization. *Conclusion*. The authors discuss thromboembolic disease and tumor metastasis roles in a cancer patient with SVC syndrome. Thromboembolism in a malignancy context is a challenging clinical entity. A multifactorial perspective of the thrombotic disease is warranted to approach thromboembolism risk and stratify patients suitable to receive adequate anticoagulant prophylaxis and targeted therapies, aiming to improve clinical prognosis.

## 1. Introduction

SVC syndrome results from the obstruction of blood flow through the SVC into the right atrium. Obstruction can be caused by invasion or external compression of the SVC by adjacent pathologic processes involving the lung, lymph nodes, and other mediastinal structures or by thrombosis of blood within the SVC [1]. In the past, infectious lesions were common causes, but nowadays malignancy and the use of intravascular devices and cardiac pacemakers have become the main causes [1, 2]. Lung cancer and lymphoma are responsible for more than 80% of SVC syndrome cases that are caused by malignancy [1, 2]. In contrast to external compression caused by malignancy, intraluminal metastatic obstruction causing thrombosis of the SVC is rare.

A relationship between cancer and thrombosis has been recognized over the years, leading to the consideration that cancer might be a prothrombotic disease [3]. Cancer is associated with an increased risk of venous thromboembolism (VTE), depending on tumor-related factors (histological type and primary tumor site; stage of disease; and time of the event from diagnosis), patient-related factors (age; comorbid conditions as obesity or pulmonary disease; thrombophilia; and immobilization), and treatment-related factors (central venous catheters; recent major surgery; chemotherapy; and transfusions) [4].

The authors describe the case of a patient diagnosed with colon carcinoma presented as SVC syndrome, highlighting the multifactorial pathophysiological association between both clinical entities.

## 2. Case Report

**Figure 1:**
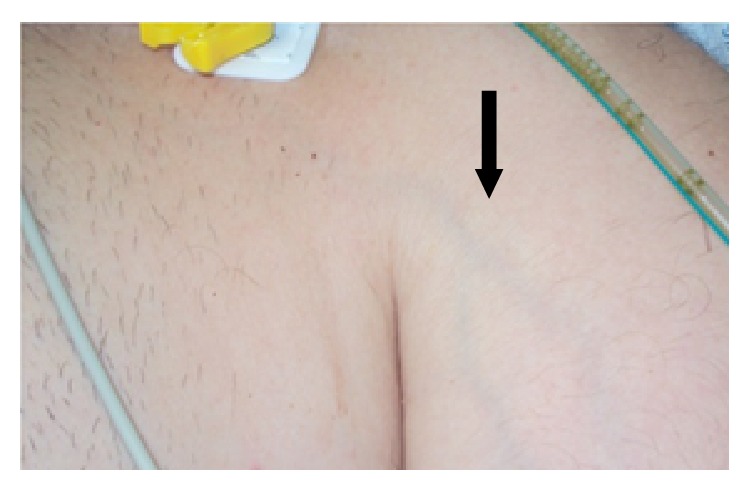
Physical examination: upper left limb edema with visible collateral veins above left clavicle and in the left arm.

A 58-year-old man with a week history of mild dyspnea and chest pain, diffuse abdominal pain with back irradiation, fatigue, and anorexia was referred to our hospital with sudden swelling and pain of the head, neck, and upper left limb. He was obese (Grade 1, BMI 34,5 Kg/m^2^) and had no other relevant medical history known. On physical examination, he was oriented, hemodynamically stable, and acyanotic and had face, neck, and upper left limb painful edema with visible collateral veins above left clavicle and diffuse abdominal pain, without signs of peritoneal irritation. Heart and lung examination revealed no abnormalities (Figure 1).

**Figure 2:**
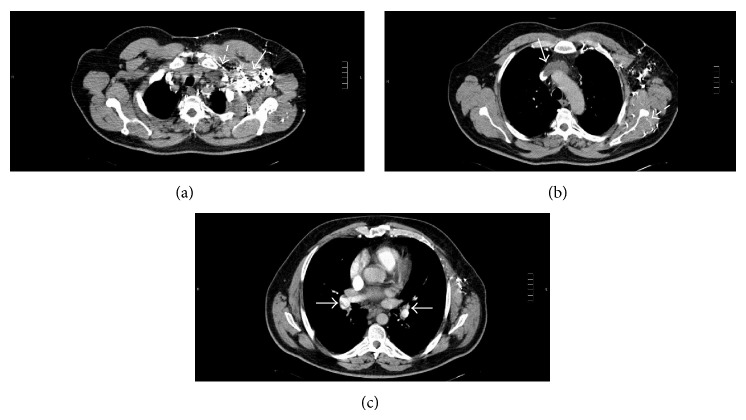
(a) Chest computed tomography angiogram. Thrombotic obstruction of the left brachiocephalic vein (dashed arrow) with multiple collateral vessels near the left clavicle and in the upper left limb (full arrow). (b) Chest computed tomography angiogram. Extension of the left brachiocephalic vein thrombus into the superior vena cava (full arrow). Collateral vessels are seen adjacent to the left clavicle and in the upper left limb (dashed arrow). (c) Chest computed tomography angiogram. Arterial thromboemboli in the pulmonary arteries (full arrows).

Routine laboratory tests showed mild normocytic anemia (hemoglobin 11,5 g/dL, 12–16 g/dL; MCV 84,7 fL, 80–100 fL) and a slightly elevation of LDH (256 U/L, 0–248 U/L) and RCP (5,23 mg/dL, 0–0,5 mg/dL). The patient had elevated D-dimers (17,51 *μ*g/mL, 0–0,60 *μ*g/mL) with normal fibrinogen level (2,3 g/L, 2–5 g/L) and normal prothrombin time (PT 14,6 s, control 13,6 s), activated partial thromboplastin time (APTT 29,5, control 28 s), and platelets (316 G/L, 150–400 G/L). He presented with hypoxemia (PaO_2_ 76 mmHg, SaO_2_ 92%) in arterial blood gas analysis. Chest computed tomography angiogram showed left axillary-subclavian thrombosis with complete obstruction of the left brachiocephalic vein and superior vena cava thrombosis and thromboemboli in the right and left pulmonary arteries (Figures 2(a), 2(b), and 2(c)).

Diagnostic investigation to evaluate the underlying SVC syndrome and pulmonary thromboembolism (PE) etiology was proceeded while anticoagulant treatment with therapeutic dosage of enoxaparin was empirically initiated. Screening for thrombophilia showed elevated Von Willebrand factor 201% (70–120%), normal factor VIII (141%, 50–150%), borderline levels of antithrombin III (80%, 80–120%), and proteins C and S normal levels with baseline-normal response to activated protein C (APC); lupus anticoagulant and anticardiolipin antibodies were negative. Molecular studies showed absence of factor V Leiden and prothrombin GA 20210 mutation, PAI 4G/4G homozygosity, PIA 1/PIA 2 GPIIIA heterozygosity, and MTHFR C677T Ala/Val heterozygosity, with normal serum homocysteine. There was no evidence of paraproteinemia. Doppler imaging of lower limbs revealed no alterations and Doppler imaging of the left upper limb showed thrombosis of the axillary and subclavian veins. Echocardiogram showed severe enlargement of the right ventricle with discrete anterior pericardium effusion, with no intracardiac thrombi.

**Figure 3:**
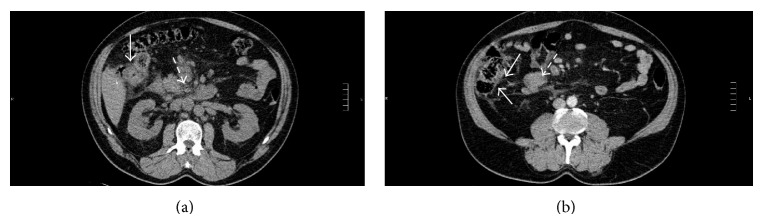
(a) Abdominal computed tomography. Circumferential thickening of the ascending colon wall (full arrow), multiple mesenteric lymphadenopathy, and thickening of the mesenteric fat (dashed arrow). (b) Abdominal computed tomography. Stranding of the pericolic fat of the ascending colon (full arrows) and lymphadenopathy conglomerate adjacent to the right anterior pararenal fascia (dashed arrow).

The patient maintained hemodynamic stability and pain control with analgesia. After 4 days of anticoagulant treatment, the patient started abundant hematochezia, needing transfusion of several unities of red blood cell (RBC) and fresh frozen plasma (FFP) (indirect Coombs test was negative). Abdominal computed tomography was performed and showed circumferential thickening of the ascending colon wall; lumbar, aortic, and mesenteric lymphadenopathy with an adenopathy conglomerate near the ascending colon and thickening of the mesenteric fat, without evident abdominal vascular thrombosis, were shown by the angiogram (Figures 3(a) and 3(b)).

**Figure 4:**
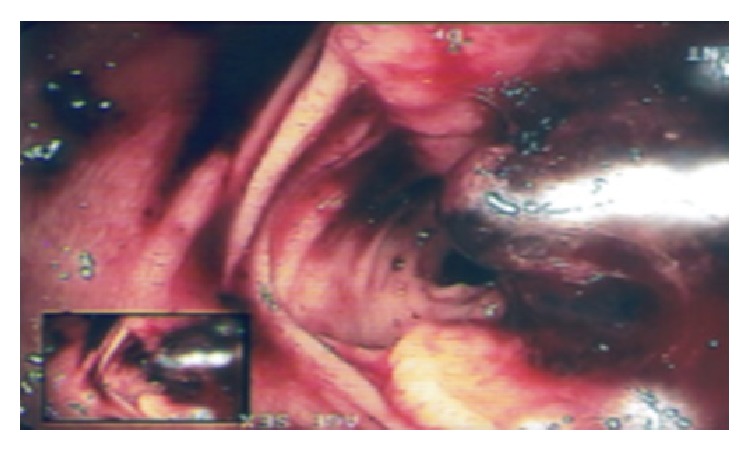
Colonoscopy. Friable and bleeding tumor in the colon hepatic flexure, obstructing the lumina.

Colonoscopy revealed a friable and bleeding neoplastic formation in the colon hepatic flexure, obstructing 3/4 of the lumina (Figure 4).

The patient was submitted to urgent surgical resection of the right colon and the retroperitoneal mass; a massive hemoperitoneum was drained (3 L). A superior vena cava tissue biopsy was not possible to obtain.

**Figure 5:**
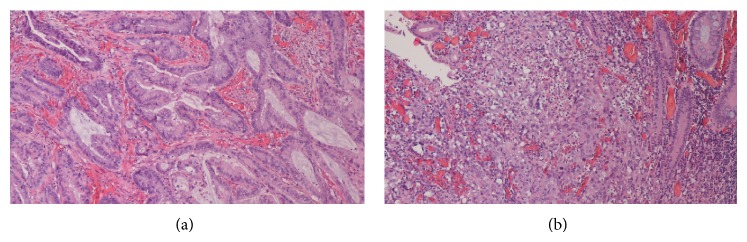
(a) Colon hepatic flexure tumor biopsy. Moderately differentiated adenocarcinoma of the colon hepatic flexure (hematoxylin and eosin staining, ×100). (b) Ascending colon tumor biopsy. Undifferentiated carcinoma of the ascending colon (hematoxylin and eosin staining, ×100).

Immunohistological examination revealed 2 synchronous tumors: a 3 cm diameter moderately differentiated adenocarcinoma of the colon hepatic flexure and a 5 cm diameter undifferentiated carcinoma of the ascending colon, with a retroperitoneal malignant adenopathy mass of 7 cm diameter, and multiple venolymphatic emboli in the tumor stroma and mesenteric metastatic disease (T4N2M1) (Figures 5(a) and 5(b)).

Three days after surgery, the patient presented low platelet count (74 G/L), prolonged PT (>6 s than control), a strong increase of D-dimers (74,94 *μ*g/mL), and decreased fibrinogen (<1 g/L). The patient maintained abdominal bleeding and significant hemoperitoneum was again drained 7 days after the first surgery. Disseminated intravascular coagulopathy (DIC) diagnosis was established and the patient was treated with prophylactic enoxaparin, corticotherapy, FFP and RBC unities, and fibrinogen concentrate. Large spectrum antibiotics were given and chemotherapy was not started. Hemostatic and coagulation parameters were continuously monitored. The patient died 40 days after the hospitalization, with an abdominal septic shock caused by multidrug-resistant* Acinetobacter baumannii* and methicillin-resistant* Staphylococcus aureus* and a DIC.

## 3. Discussion

SVC syndrome is a historic clinical entity commonly associated with malignant conditions [1, 2]. Cancer seems to cause an unbalance between coagulation and fibrinolytic systems, leading to a hypercoagulable state [3]. Horsted et al. reported an estimated annual incidence rate of VTE between 0,5 and 20%, depending on the cancer type and background risk [5]. Pancreas, haematological, brain, and lung cancers are associated with greater risk of VTE, with colorectal cancer patients having a relative risk of incidence of VTE of 3,93, when compared with the general population [5]. Approximately 3% of the patients with colorectal cancer develop VTE within 2 years, being the incidence highest in the first months after the cancer diagnosis, in advanced cancer stages and in the presence of medical comorbidities [6]. By the time the patient was diagnosed with SVC syndrome and PE, a diagnostic workup was carried out in order to find the underlying disease behind the major thrombotic event that led the patient to the hospital. Clinical thromboembolism can be the first manifestation of a malignancy, appearing even before the cancer has become symptomatic [7, 8], as was the patient's case. The patient had a metastatic colon cancer by the time of the diagnosis without having any previous symptom to the SVC syndrome and the PE, possibly translating a biologically more aggressive tumor. Besides, he was diagnosed with a metastatic colon adenocarcinoma, histological subtype believed to be associated with higher risk of developing VTE [4].

Scarce previous published data reported cases of intraluminal tumor metastasis causing SVC obstruction, especially from colorectal cancer. Alzand et al. reported a case suggesting metastasis of colon carcinoma into the SVC leading to intraluminal obstruction; immunohistochemical staining of the intraluminal tumor that was obstructing and invading SVC wall was strongly suggestive of a primary tumor originating from the colon [9]. Bockorny et al. described a case of a SVC syndrome due to an extensive mass in the SVC, in which intravascular biopsy showed metastatic colon cancer [10]. The patient could eventually have a SVC intraluminal metastasis, as the ascending colon is a retroperitoneal structure surrounded by the lumbar and vertebral veins branches, draining into the azygos system and subsequently into the SVC. Not only the patient had the ascending colon and colon hepatic flexure tumors, he also had a retroperitoneal malignant adenopathy conglomerate that could invade one of these branches, sending intraluminal metastasis into the SVC. Autopsy and histological examination of SVC would have been essential to confirm whether intraluminal obstruction was caused by colon tumor metastasis or by venous thromboembolic disease.

Multiple underlying pathologic coexisting mechanisms confer VTE risk, and consequently SVC venous thrombosis, in a cancer patient. Indeed, in a cancer patient, each of the 3 features of the Virchow triad that predispose to thrombus formation are abnormal: viscosity and stasis are increased, slowing blood flow; platelet aggregation and activation and procoagulant factors are increased together with decreased fibrinolytic and anticoagulant factors; under the influence of inflammatory cytokines, endothelial cells can become prothrombotic, conducting to a dysfunctional endothelium [3, 4, 7]. The patient presented an elevated Von Willebrand factor that can be attributable to tumor activity [11], whereas the antithrombin III level near the lower limit of the normal range may represent an effect of enoxaparin.

Thrombotic events in cancer patients usually manifest as classical deep-venous thrombosis of the limbs and PE, but thrombosis of the vena cava and visceral circulation, DIC, and thrombotic microangiopathy can also develop [3]. There is substantially more data on venous thromboembolism than on arterial thrombosis in malignancy, underlining that arterial thrombosis is less frequent than venous one in cancer. However, arterial thrombosis and cancer association has been recognized through time; the underlying mechanisms are still not completely clear [11, 12]. Arterial thrombosis clinical manifestations can include localized arterial occlusion, thrombotic thrombocytopenic purpura, and DIC. Besides SVC thrombosis, the patient presented arterial thromboemboli in the pulmonary arteries and developed DIC after surgery.

“DIC is characterized by widespread intravascular activation of coagulation (leading to intravascular fibrin deposition) and simultaneous consumption of coagulation factors and platelets (potentially resulting in bleeding)” [13]. According to previous published data, DIC was diagnosed in 7% of the patients with solid tumors and was more frequent in advanced-stage cancer [14, 15]. DIC has generally a less severe presentation in cancer when compared to the types of DIC associated with sepsis and trauma [13]. Not only had the patient an advanced cancer, but he was also submitted to a major abdominal surgery; he was immobilized in bed for a significant period and developed sepsis after the procedure, all the factors contributing to a more fulminant outcome. It has been reported by Prandoni et al. that cancer patients with venous thrombosis have higher risk of recurrent thromboembolism and major bleeding during anticoagulant therapy than those without malignancy [16]. This patient was treated with therapeutic enoxaparin, for SVC syndrome and PE, for four days before starting bleeding. On the other hand, it has been shown that the incidence of VTE and mortality is higher in hospitalized cancer patients who received RBC transfusion compared with hospitalized cancer patients that were not transfused [17]. The patient was transfused with several RBC and FFP unities during the hospitalization, which might favored the DIC development. Albeit intravascular devices are known to induce thrombotic complications, the patient never had central venous catheters before the main thrombotic event and SVC intravascular stenting was not attempted given the patient coagulopathy and initial absence of life-threatening symptoms with a posterior diagnosis of malignancy during hospitalization.

In addition to the tumor- and treatment-related factors, the patient also had some molecular alterations contributing to an abnormal hemostasis and tumor aggressiveness, although he never had a reported prior history of thromboembolism. 4G/4G genotype is associated with a higher risk of thrombosis [18, 19]; GPIIIA PlA2 polymorphism has been associated with thrombosis [20, 21]; MTHFR 677T variant allele is associated with cancer risk, influencing the clinical phenotype and the interaction with other environmental factors [22, 23]. MTHFR C677T polymorphism has also been associated with VTE risk [24]. Moreover, the patient was obese and obesity is a significant risk factor for VTE and arterial thrombosis [25, 26].

SVC syndrome is a clinically striking entity with distinct pathophysiological mechanisms in a malignancy context. Cancer patients have multiple risk factors for thromboembolic disease that vary along with patient-, cancer-, and treatment-related features, having therapeutic and prognostic implications. An individualized clinical approach is important to pursue the accurate diagnosis of the underlying disorder causing thromboembolism in cancer. A multifactorial perspective of the thrombotic disease is warranted to estimate thromboembolism risk and stratify patients suitable to receive adequate anticoagulant prophylaxis and targeted therapies.

## References

[1] Wilson L. D., Detterbeck F. C., Yahalom J. (2007). Superior vena cava syndrome with malignant causes. *The New England Journal of Medicine*.

[2] Rice T. W., Rodriguez R. M., Light R. W. (2006). The superior vena cava syndrome: clinical characteristics and evolving etiology. *Medicine*.

[3] Lip G. Y., Chin B. S., Blann A. D. (2002). Cancer and the prothrombotic state. *Lancet Oncology*.

[4] Streiff M. B. (2013). Association between cancer types, cancer treatments, and venous thromboembolism in medical oncology patients. *Clinical Advances in Hematology and Oncology*.

[5] Horsted F., West J., Grainge M. J. (2012). Risk of venous thromboembolism in patients with cancer: a systematic review and meta-analysis. *PLoS Medicine*.

[6] Alcalay A., Wun T., Khatri V. (2006). Venous thromboembolism in patients with colorectal cancer: incidence and effect on survival. *Journal of Clinical Oncology*.

[7] Blann A. D., Dunmore S. (2011). Arterial and venous thrombosis in cancer patients. *Cardiology Research and Practice*.

[8] Sørensen H. T., Mellemkjær L., Steffensen F. H., Olsen J. H., Nielsen G. L. (1998). The risk of a diagnosis of cancer after primary deep venous thrombosis or pulmonary embolism. *The New England Journal of Medicine*.

[9] Alzand B. S. N., Geyik Z., Dennert R., Cheriex E. C. (2009). Superior vena cava syndrome as a complication of colon carcinoma. *International Journal of Cardiology*.

[10] Bockorny M., Kourelis T., Bockorny B. (2012). Superior vena cava syndrome caused by colon adenocarcinoma metastasis: a case report and review of literature. *Connecticut Medicine*.

[11] Uchiyama T., Matsumoto M., Kobayashi N. (1990). Studies on the pathogenesis of coagulopathy in patients with arterial thromboembolism and malignancy. *Thrombosis Research*.

[12] Sack G. H., Levin J., Bell W. R. (1977). Trousseau's syndrome and other manifestations of chronic disseminated coagulopathy in patients with neoplasms: clinical, pathophysiologic, and therapeutic features. *Medicine*.

[13] Levi M. (2009). Disseminated intravascular coagulation in cancer patients. *Best Practice and Research: Clinical Haematology*.

[14] Sallah S., Wan J. Y., Nguyen N. P., Hanrahan L. R., Sigounas G. (2001). Disseminated intravascular coagulation in solid tumors: clinical and pathologic study. *Thrombosis and Haemostasis*.

[15] Colman R. W., Rubin R. N. (1990). Disseminated intravascular coagulation due to malignancy. *Seminars in Oncology*.

[16] Prandoni P., Lensing A. W. A., Piccioli A. (2002). Recurrent venous thromboembolism and bleeding complications during anticoagulant treatment in patients with cancer and venous thrombosis. *Blood*.

[17] Khorana A. A., Francis C. W., Blumberg N., Culakova E., Refaai M. A., Lyman G. H. (2008). Blood transfusions, thrombosis, and mortality in hospitalized patients with cancer. *Archives of Internal Medicine*.

[18] Balta G., Altay C., Gurgey A. (2002). PAI-1 gene 4G/5G genotype: a risk factor for thrombosis in vessels of internal organs. *The American Journal of Hematology*.

[19] Seguí R., Estellés A., Mira Y. (2000). PAI-1 promoter 4G/5G genotype as an additional risk factor for venous thrombosis in subjects with genetic thrombophilic defects. *British Journal of Haematology*.

[20] Weiss E. J., Bray P. F., Tayback M. (1996). A polymorphism of a platelet glycoprotein receptor as an inherited risk factor for coronary thrombosis. *The New England Journal of Medicine*.

[21] Ardissino D., Mannucci P. M., Merlini P. A. (1999). Prothrombotic genetic risk factors in young survivors of myocardial infarction. *Blood*.

[22] Kim Y.-I. (2009). Role of the MTHFR polymorphisms in cancer risk modification and treatment. *Future Oncology*.

[23] Toffoli G., de Mattia E. (2008). Pharmacogenetic relevance of MTHFR polymorphisms. *Pharmacogenomics*.

[24] Zhang P., Gao X., Zhang Y. (2014). Association between MTHFR C677T polymorphism and venous thromboembolism risk in the Chinese population: a meta-analysis of 24 case-controlled studies. *Angiology*.

[25] Stein P. D., Beemath A., Olson R. E. (2005). Obesity as a risk factor in venous thromboembolism. *The American Journal of Medicine*.

[26] Borch K. H., Nyegaard C., Hansen J.-B. (2011). Joint effects of obesity and body height on the risk of venous thromboembolism: the tromsø study. *Arteriosclerosis, Thrombosis, and Vascular Biology*.

